# The Chloroplast Genome of *Hyoscyamus niger* and a Phylogenetic Study of the Tribe Hyoscyameae (Solanaceae)

**DOI:** 10.1371/journal.pone.0098353

**Published:** 2014-05-22

**Authors:** M. Virginia Sanchez-Puerta, Cinthia Carolina Abbona

**Affiliations:** 1 Facultad de Ciencias Exactas y Naturales, IBAM-CONICET and Facultad de Ciencias Agrarias, Universidad Nacional de Cuyo, Chacras de Coria, Mendoza, Argentina; 2 IBAM-CONICET and Facultad de Ciencias Agrarias, Universidad Nacional de Cuyo, Chacras de Coria, Mendoza, Argentina; Naval Research Laboratory, United States of America

## Abstract

The tribe Hyoscyameae (Solanaceae) is restricted to Eurasia and includes the genera *Archihyoscyamus, Anisodus, Atropa, Atropanthe, Hyoscyamus, Physochlaina, Przewalskia* and *Scopolia*. Even though the monophyly of Hyoscyameae is strongly supported, the relationships of the taxa within the tribe remain unclear. Chloroplast markers have been widely used to elucidate plant relationships at low taxonomic levels. Identification of variable chloroplast intergenic regions has been developed based on comparative genomics of chloroplast genomes, but these regions have a narrow phylogenetic utility. In this study, we present the chloroplast genome sequence of *Hyoscyamus niger* and make comparisons to other solanaceous plastid genomes in terms of gene order, gene and intron content, editing sites, origins of replication, repeats, and hypothetical open reading frames. We developed and sequenced three variable plastid markers from eight species to elucidate relationships within the tribe Hyoscyameae. The presence of a horizontally transferred intron in the mitochondrial *cox1* gene of some species of the tribe is considered here a likely synapomorphy uniting five genera of the Hyoscyameae. Alternatively, the *cox1* intron could be a homoplasious character acquired twice within the tribe. A homoplasious inversion in the intergenic plastid spacer *trnC-psbM* was recognized as a source of bias and removed from the data set used in the phylogenetic analyses. Almost 12 kb of plastid sequence data were not sufficient to completely resolve relationships among genera of Hyoscyameae but some clades were identified. Two alternative hypotheses of the evolution of the genera within the tribe are proposed.

## Introduction

The family Solanaceae consists of more than 2,700 species, including several economically important crops and ornamentals. The genus *Hyoscyamus* includes some of the most important medicinal plants belonging to the Solanaceae [1], [2]. Species of *Hyoscyamus* and related genera are well known as a natural source of tropane alkaloids such as hyoscyamine, scopolamine, and tropine, which have medicinal, hallucinogenic and poisonous properties [2], [3], [4]. The tribe Hyoscyameae is largely restricted to Eurasia and includes the genera *Archihyoscyamus*, *Anisodus*, *Atropa*, *Atropanthe*, *Hyoscyamus*, *Physochlaina*, *Przewalskia* and *Scopolia* [5], [6], [7], [8]. Albeit the delimitation of the tribe has been questioned based on secondary chemistry [1], [9], today the monophyly of the tribe Hyoscyameae is strongly supported based on molecular data [5], [10]. What remains unclear, however, are the relationships among the taxa of the tribe.

Morphological characters [1], alkaloid biosynthetic pathways [3], [4], cytological features [7], and a few molecular markers [5], [6], [10], [11] have been used to elucidate the phylogeny of the tribe Hyoscyameae. The resulting tree topologies based on different types of data are incongruent and the evolutionary relationships among the genera remain controversial to date. However, previous studies agreed on the monophyly of the genera *Anisodus*, *Hyoscyamus*, and *Physochlaina*, but disagreed on the monophyly of *Scopolia* [5], [10]. *Archihyoscyamus*, *Atropanthe* and *Przewalskia* are monotypic genera, each containing a single species. Phylogenetic studies based on plastid molecular markers showed that *Physochlaina*, *Przewalskia* and *Scopolia* formed a well-supported clade [5], [10], but the relationships of this clade with respect to *Archihyoscyamus*, *Hyoscyamus*, *Anisodus* and *Atropanthe* remain unresolved.

More than 260 chloroplast genomes are available from different species of land plants and ten of those belong to the angiosperm family Solanaceae: *Atropa belladonna, Capsicum annuum, Datura stramonium*, four species of *Nicotiana*, and three of *Solanum* [12], [13], [14], [15]. Within the tribe Hyoscyameae, only one chloroplast genome has been sequenced [16], belonging to *Atropa belladonna*. Chloroplast intergenic regions have been useful markers to explore phylogenetic relationships of plants and algae at different taxonomic levels [17], [18]. However, appropriate plastid regions cannot be developed without the chloroplast genome sequence of representative lineages of the group under study because different plant groups do not share the same variable plastid markers at low taxonomic levels [17], [18], [19], [20].

Here, we present the complete chloroplast genome sequence for *Hyoscyamus niger* and a phylogenetic study of the tribe Hyoscyameae based on novel plastid markers developed by comparative genomics. We also include a thorough comparison to other solanaceous plastid genomes. The goals of this study are: 1) to compare the chloroplast genomes of ten solanaceous species in terms of genome organization, gene and intron content, RNA editing pattern, and origin of replication, 2) to identify highly variable intergenic regions that would aid in understanding relationships of the genera within the tribe Hyoscyameae; and 3) to sequence these rapidly evolving plastid sequences from members of the tribe Hyoscyameae and test their usefulness to unveil phylogenetic relationships.

## Materials and Methods

### Plant material and DNA extraction

Seeds of *Atropanthe sinensis* (NBG944750119), *Hyoscyamus niger* (NBGA04750027), *H. aureus* (NBG914750063), *H. turcomanicus* (NBG904750014), *H. muticus* (NBG974750072), *Physochlaina orientalis* (NBG944750045), and *P. physaloides* (NBG924750021) were obtained from the Nijmegen Botanical Garden (The Netherlands). Total genomic DNA was extracted from leaves using a cetyl-trimethyl-ammonium-bromide (CTAB) DNA-extraction protocol [21]. In addition, DNA samples of *Anisodus tanguticus* (RGO2003-083b) and *Przewalskia tangutica* (RGO2003-090) were donated by Richard Olmstead (University of Washington).

### Genome sequencing and assembly

Total genomic DNA from *H. niger* was sequenced at the Beijing Genomics Institute (BGI) with Illumina Hiseq2000 Sequencing Technology (Illumina Inc.). This produced about 6.86 Gbp (equivalent to 70 million reads) of clean paired-end reads of 80 bp. The DNA library had a mean size of 888 bp (SD = 2934 bp). Paired-end reads were assembled using the Velvet assembler 1.2.03 (Daniel Zerbino, European Bioinformatics Institute, UK). Based on the difference in read depth, nuclear (read depth <5), mitochondrial (read depth between 20 and 130), and chloroplast (read depth >150) contigs were separated. Genome assemblies and read-pair mapping patterns were visually inspected using Consed 16.0 [22]. Based on read-pair information, chloroplast contigs were connected into a single contig representing the circular map of the cpDNA. The resulting chloroplast genome was iteratively compared against all reads to identify errors until no errors remained, that is, until the high quality read depth and paired-end read depth was as expected at each base and the high quality mismatches were very low or zero.

### Genome annotation

The chloroplast genome of *H. niger* was annotated using DOGMA [23]. Graphical genome maps were generated using OGDraw software [24]. The annotated plastid genome sequence of *H. niger* is available from GenBank (KF248009). Using the software ORF (MolGen, University of Groningen, Netherlands), we annotated ORFs (open reading frames) with unknown functions. Information on tandem repeats was obtained using the Tandem Repeats Finder program [25], with alignment parameters set as 2, 7, 7 for match, mismatch and indels, respectively. The maximum period size and minimum alignment score were 500 and 50, respectively. Sites of RNA editing in protein-coding genes of *Hyoscyamus niger* were predicted using PREP-Mt [26] with a cutoff value of 0.8.

### Comparison to other solanaceous chloroplast genomes

Pairwise analyses between six solanaceous chloroplast genomes were done with mVISTA program [27] in Shuffle-LAGAN mode and with the BLAST from NCBI [28]. The whole genome identity between *H. niger* and each of the other solanaceous cpDNAs was calculated by VISTA. To compare predicted editing sites in each cpDNA, each known coding-gene was aligned using MacClade 4.07 [29] and analyzed individually.

### Amplification and sequencing of selected chloroplast regions for phylogenetic studies

Based on pairwise comparisons of complete sequences of the chloroplast genomes from *Hyoscyamus niger, Atropa belladonna*, *Capsicum annuum, Datura stramonium, Nicotiana tabacum* and *Solanum tuberosum* we identified three rapidly-evolving intergenic regions that could be useful in resolving relationships within the tribe Hyoscyameae. We designed three primer pairs to amplify those regions by PCR from eight species of the tribe Hyoscyameae (Table S1). An intergenic region of 849–984 bp between the genes *rps16* and *trnQ* was amplified with the primers Ncprps16 (5′ TGATGTATAAACACCATAATC 3′) and NcptrnQ (5′ TTCTCTACCTCCTAAATTAG 3′). Using the primer pair Ncpycf3 (5′ CATACTAATGTAGTAGTATAGG 3′) and Ncprps4 (5′ CTTAAACCTCAGACTGAAAC 3′), a region of 796–987 bp between the genes *ycf3* and *rps4* was amplified. To amplify the ∼650 bp region between the genes *ndhF* and *rpl32*, the primers NcpndhF (5′ ATTCACCGGATCTTACCTCT 3′) and Ncprpl32 (5′ AGCTAATAGTGTCCTCCTCAA 3′) were used. PCR conditions included initial denaturing at 94°C for 2 min; followed by 35 cycles of 94°C for 40 sec, 50°C for 45 sec, and 72°C for 1 min; followed by a final extension at 72°C for 8 min. PCR products were sequenced using an ABI 3730 (Applied Biosystems).

### Sequence and phylogenetic analyses

Sequences were aligned manually with MacClade 4.0 [29]. Phylogenetic analyses were performed on individual and concatenated data sets of the three intergenic regions identified in this study (*rps16-trnQ, ycf3-rps4, ndhF-rpl32*) and seven previously reported regions (*trnL-F*, *trnC-psbM*, *psbA-trnH*, *rps16-trnK, rbcL, ndhF* and *atpB*) retrieved from GenBank. GenBank accession numbers of angiosperm sequences are listed in Table S1. Phylogenetic analyses were performed individually for each region and then all plastid markers where concatenated in a single data set. Maximum likelihood analyses were performed with Garli 0.951 [30] under the General Time Reversible model with parameters for invariable sites and gamma-distributed rate heterogeneity (GTR+I+Γ4; four rate categories). This substitution model was supported by hierarchical likelihood ratio tests performed using JModeltest [31]. Ten independent runs were conducted using either the automated stopping criterion or for up to 5,000,000 generations to ensure convergence to a similar topology and likelihood score. One hundred bootstrap (BS) replicates were performed. Bayesian inference using Mr. Bayes 3.1.2 [32] was run for 10^6^ generations and the average standard deviation of split frequencies was 0.01. Posterior probabilities (PP) were obtained from the Bayesian analysis. In addition, maximum parsimony (MP) analyses were performed using PAUP* [33] with 1000 bootstrap replicates.

### Alternative topology test

The approximately unbiased (AU) test [34] was used to evaluate whether a particular topology was significantly better than a specified (constrained) alternative topology. The CONSEL package [35] was used to calculate the probability value (p-value) of the AU test to assess the confidence in the comparison of unconstrained (best tree) and constrained trees. Constrained trees included: A) monophyly of intron-containing genera [*Hyoscyamus* (including *Archihyoscyamus*), *Physochlaina*, *Przewalskia* and *Scopolia*] and B) single occurrence of a 10 nt-inversion, i.e. monophyly of the genera *Anisodus* and *Przewalskia*. The most likely tree under each constraint was determined by searching for the best tree compatible with that constraint using PAUP* [33]. The site likelihoods for constrained and unconstrained trees were calculated with PAUP* and exported to CONSEL to run the AU test.

## Results and Discussion

### Hyoscyamus niger chloroplast genome

**Figure 1:**
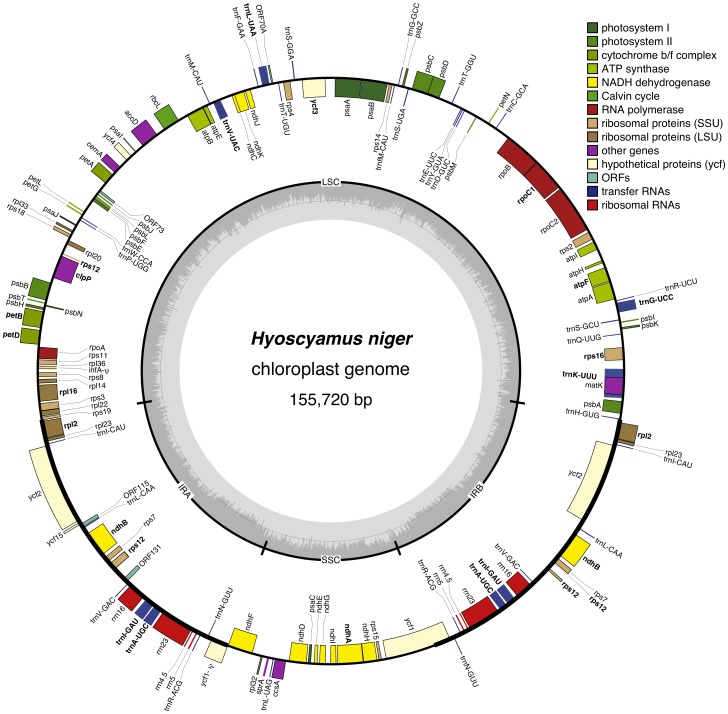
Chloroplast genome of *Hyoscyamus niger*. Large and small single copy regions (LSC, SSC) and inverted repeats (IR) are indicated. Intron-containing genes are in bold face. Genes drawn inside and outside the circle are transcribed clockwise and counterclockwise, respectively. Genes belonging to different functional groups are marked with colors. Internal circle shows the %GC content across the cpDNA. A line is shown at GC content of 50%.

Next-generation sequencing, such as Illumina Hiseq2000 technology, proved to be a fast and efficient method to sequence the organellar genome of *Hyoscyamus niger*. The chloroplast genome of *H. niger* assembled into a single circular molecule of 155,720 bp in length and had a quadripartite structure similar to that of most land plant chloroplast genomes (Figure 1). The inverted repeat (IR) and large (LSC) and small single copy (SSC) regions were 25,876, 86,105 and 17,863 bp long, respectively. The global GC-content was 37.6% (LSC: 35.6%, SSC: 31.5% and IR: 42.9%). RNA genes showed a high GC-content (55.4%), probably necessary for appropriate folding of rRNAs and tRNAs.

The genome encodes 80 protein-coding genes and conserved hypothetical chloroplast reading frames (*ycfs*), 4 rRNA and 30 tRNA genes, not counting identical copies (Table 1). Five protein-coding genes, 4 rRNAs and seven tRNAs were duplicated in the IR. The gene *infA*, which codes for a translation initiation factor, is a pseudogene in *H. niger*, tobacco, tomato and *Atropa* [14], [16], [36]. The tRNA coding capacity of the *H. niger* cpDNA (30 tRNAs) may constitute the complete set for decoding all codons in protein-coding genes through extended wobbling or superwobbling [37]. Twelve protein-coding genes [*atpF, clpP* (2 introns), *ndhA, ndhB, petB, petD, rpl16, rpl2, rpoC1, rps12* (2 introns), *rps16* and *ycf3* (2 introns)] and six tRNA genes (*trnA-UGC, trnG-UCC, trnI-GAU, trnK-UUU, trnL-UAA*, and *trnV-UAC*) contained introns. Nineteen of them were cis-spliced Group II introns, except for intron 1 in the *rps12* gene, which was trans-spliced, and the intron found in *trnL-UAA*, which was a Group I intron. Two atypical start codons were predicted for plastid genes of *H. niger*; ACG in *psbL* and *ndhD*, and GTG in *rps19*. Previous studies [14], [16], [38] indicated that RNA editing modifies the start codon ACG to AUG in the chloroplast genes *psbL* and *ndhD* in *Nicotiana tabacum* and *Atropa belladonna* (Table 2).

**Table 1:** Genes identified in the chloroplast genome of *Hyoscyamus niger*.

Photosynthesis-related	
Photosystem I	*psaA, psaB, psaC, psaI, psaJ, * ***ycf3*** *, ycf4*
Photosystem II	*psbA, psbB, psbC, psbD, psbE, psbF, psbH, psbI, psbJ, psbK, psbL, psbM, psbN, psbT, psbZ (ycf9)*
Cytochrome b_6_f complex	*petA, * ***petB*** *, * ***petD*** *, petG, petL, petN (ycf6)*
NAD(P)H dehydrogenase	***ndhA*** *, * ***ndhB*** *, ndhC, ndhD, ndhE, ndhF, ndhG, ndhH, ndhI, ndhJ, ndhK*
ATP synthase	*atpA, atpB, atpE, * ***atpF*** *, atpH, atpI*
Calvin cycle	*rbcL*
Ribosomal proteins	
Large subunit	***rpl2*** *, rpl14, * ***rpl16*** *, rpl20, rpl22, rpl23, rpl32, rpl33, rpl36*
Small subunit	*rps2, rps3, rps4, rps7, rps8, rps11, * ***rps12*** *, rps14, rps15, * ***rps16*** *, rps18, rps19*
RNA polymerases	*rpoA, rpoB, * ***rpoC1*** *, rpoC2*
Others	*matK, * ***clpP*** *, accD, ccsA (ycf5), cemA (ycf10), sprA*
Hypothetical proteins	*ycf1, ycf2, ycf15*
ORFs	*orf70A, orf73, orf115, orf131*
rRNAs	*rrn16, rrn23, rrn4.5, rrn5*
tRNAs	***trnA-UGC*** *, trnC-GCA, trnD-GUC, trnE-UUC, trnF-GAA, trnfM-CAU, trnG-GCC, * ***trnG-UCC*** *, trnH-GUG, trnI-CAU, * ***trnI-GAU*** *, * ***trnK-UUU*** *, trnL-CAA, * ***trnL-UAA*** *, trnL-UAG, trnM-CAU, trnN-GUU, trnP-UGG, trnQ-UUG, trnR-ACG, trnR-UCU, trnS-GCU, trnS-GGA, trnS-UGA, trnT-GGU, trnT-UGU, trnV-GAC, * ***trnV-UAC*** *, trnW-CCA, trnY-GUA*

Note: Bold face for intron-containing genes.

**Table 2:** Editing sites in chloroplast genes of Solanaceae. Predicted editing sites in *Hyoscyamus niger* plastid genes and observed editing sites in *Atropa belladonna* and *Nicotiana tabacum* cpDNAs.

Gene	Codon number	*Hyoscyamus niger (KF248009)*	*Atropa belladonna (NC_004561)*	*Nicotiana tabacum (NC_001879)*	Codon in unedited mRNA (encoded amino acid) and edited codon in mRNA (encoded amino acid)
*atpA*	264	T	T	C to U	cCc (Pro) to cUc (Leu)
*atpA*	265	C	C to U	C to U	ucC (Ser) to ucU (Ser); synonymous edit
*atpF*	31	C to U predicted	C to U	C to U	cCa (Pro) to cUa (Leu)
*ndhA*	114	C to U predicted	C to U	C to U	uCa (Ser) to uUa (Leu)
*ndhA*	189	C to U predicted	C to U	T	uCa (Ser) to uUa (Leu)
*ndhA*	358	C to U predicted	C to U	C to U	uCc (Ser) to uUc (Phe)
*ndhB*	50	C to U predicted	C to U	C to U	uCa (Ser) to uUa (Leu)
*ndhB*	156	C to U predicted	C to U	C to U	cCa (Pro) to cUa (Leu)
*ndhB*	196	C to U predicted	C to U	C to U	Cau (His) to Uau (Tyr)
*ndhB*	204	C to U predicted	C to U	C to U	uCa (Ser) to uUa (Leu)
*ndhB*	246	C to U predicted	C to U	C to U	cCa (Pro) to cUa (Leu)
*ndhB*	249	C to U predicted	C to U	C to U	uCu (Ser) to uUu (Phe)
*ndhB*	277	C to U predicted	C to U	C to U	uCa (Ser) to uUa (Leu)
*ndhB*	279	C to U predicted	C to U	C to U	uCa (Ser) to uUa (Leu)
*ndhB*	494	C to U predicted	C to U	C to U	cCa (Pro) to cUa (Leu)
*ndhD*	1	C to U predicted	C to U	C to U	aCg (Thr) to aUg (Met); start codon
*ndhD*	128	C to U predicted	C to U	C to U	uCa (Ser) to uUa (Leu)
*ndhD*	200	T	T	C to U	uCa (Ser) to uUa (Leu)
*ndhD*	225	T	T	C to U	uCg (Ser) to uUg (Leu)
*ndhD*	293	C to U predicted	C to U	T	uCa (Ser) to uUa (Leu)
*ndhD*	433	C to U predicted	C to U	C to U	uCa (Ser) to uUa (Leu)
*ndhD*	437	C to U predicted	C to U	C to U	uCa (Ser) to uUa (Leu)
*ndhF*	97	C to U predicted	C to U	C to U	uCa (Ser) to uUa (Leu)
*ndhG*	17	C to U predicted	C to U	C to U	uCg (Ser) to uUg (Leu)
*petB*	204	C to U predicted	C to U	C to U	cCa (Pro) to cUa (Leu)
*psbE*	72	C to U predicted	T	C to U	cCa (Pro) to cUa (Leu)
*psbL*	1	C to U predicted	C to U	C to U	aCg (Thr) to aUg (Met); start codon
*rpl20*	103	C to U predicted	C to U	C to U	uCa (Ser) to uUa (Leu)
*rpoA*	277	C to U predicted	C to U	C to U	uCa (Ser) to uUa (Leu)
*rpoC1*	21	C to U predicted	C to U	C to U	uCa (Ser) to uUa (Leu)
*rpoC2*	1248	C to U predicted	C to U	C to U	uCa (Ser) to uUa (Leu)
*rpoC2*	767	C to U predicted	C	C	probably incorrect prediction
*rpoB*	113	C to U predicted	C to U	C to U	uCu (Ser) to uUu (Phe)
*rpoB*	158	C to U predicted	C to U	C to U	uCa (Ser) to uUa (Leu)
*rpoB*	184	C to U predicted	C to U	C to U	uCa (Ser) to uUa (Leu)
*rpoB*	667	C to U predicted	C to U	C to U	uCu (Ser) to uUu (Phe)
*rpoB*	809	C to U predicted	C to U	T	uCa (Ser) to uUa (Leu)
*rps2*	45	C to U predicted	C to U	C to U	aCa (Thr) to aUa (Ile)
*rps2*	83	C to U predicted	C to U	C to U	uCa (Ser) to uUa (Leu)
*rps14*	27	C to U predicted	C to U	C to U	uCa (Ser) to uUa (Leu)
*rps14*	50	T	T	C to U	cCa (Pro) to cUa (Leu)
Total editing sites		36	35	37	

Note: T, the nucleotide thymine (T) is present at the DNA level and no editing is required; C, the nucleotide cytidine is present at the DNA level but no editing is observed or predicted; C to U, the cytidine found at the DNA level is edited to uridine in the mRNA. The genes that are no listed are not edited.

Four ORFs longer than 150 bp and with unknown functions were detected in *H. niger* cpDNA (ORF70A, ORF73, ORF115, ORF131; Table 1). All four ORFs were present in at least one other solanaceous chloroplast genome but showed frameshift mutations, suggesting that they are unlikely to encode functional proteins. Tandem Repeat Finder recognized 24 repeats of 9–34 nt long. The total length of tandem repeats in *H. niger* cpDNA was 1,416 bp, similar to that of tobacco and tomato chloroplast genomes [13].

### Comparisons of cpDNAs in Solanaceae

Ten chloroplast genomes from five different genera within the Solanaceae have been sequenced [12], [13], [14], [15]. The chloroplast genome of *H. niger* was highly similar to others within the family, with 97.9%, 97.0%, 96.9%, 96.5%, and 96.1% identity to *Atropa belladonna, Datura stramonium, Nicotiana tabacum, Solanum tuberosum*, and *Capsicum annuum*, respectively. A detailed comparison between *H. niger* and *A. belladonna* plastid genomes indicated that the identity varied across the genome, showing 99.32% identity in the IR, and 98.78%, and 94.88% identity in coding and intergenic regions outside of the IR, respectively. Gene conversion is known to occur between the two IR [39], [40], which could be responsible for the lower mutation rate and higher GC-content in the IR.

The following features were highly conserved in plastid genomes across the family Solanaceae: genome organization, genome size (155,296–156,781 bp), gene content (80 protein-coding genes and 34 RNA genes), gene order, intron type and content (20 group II and 1 group I introns), intron locations, and overall GC-content (37–37.9%). In contrast, unknown ORFs identified in each chloroplast genome were not conserved across the Solanaceae. Two replication origins have been experimentally mapped to the inverted repeat in tobacco [41] and both features were highly similar in the chloroplast genomes of *Petunia* [41], *Atropa* [16] and *H. niger* (this study): 1) *Ori*A, a 82 bp region encompassing 2 direct repeats followed by a stem-loop forming structure within the *trnI-GAU* intron, and 2) *Ori*B, a 243 bp region including a stem-loop forming structure and direct repeats within the *ycf1* gene.

### RNA editing in chloroplast-encoded genes in Solanaceae

RNA editing in plastids is a posttranscriptional process that converts the identity of cytidines to uridines in specific sites of primary transcripts of some chloroplast genes [42]. RNA editing sites across cpDNAs of angiosperms are only poorly conserved as a result of independent losses and acquisitions of editing sites in unrelated [43] and sometimes closely related [16], [44] lineages. A total of 36 editing sites were predicted *in silico* for 16 chloroplast genes of *H. niger* (Table 2). A comparison of observed editing patterns of two Solanaceae (*Atropa belladonna* and *Nicotiana tabacum*) indicated that editing sites are relatively conserved [14], [16], [38], occurring at 17 plastid genes out of 80 different protein-coding genes (Table 2). *Hyoscyamus niger* shared 34 editing sites with *A. belladonna* and 32 with *N. tabacum* (Table 2), while the three taxa shared 30 editing sites with *Solanum lycopersicum*, suggesting its presence in their common ancestor [14]. No *H. niger*-specific editing sites were predicted.

### Phylogenetic relationships within the tribe Hyoscyameae

**Figure 2:**
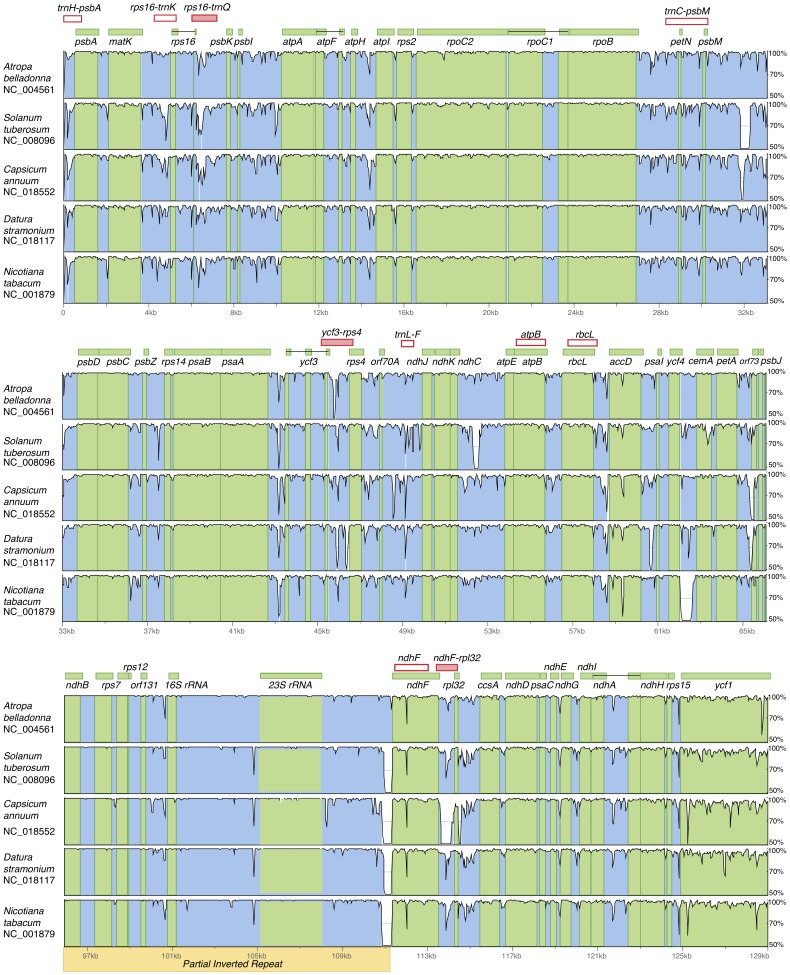
Identity plots comparing *Hyoscyamus niger* chloroplast genome to other Solanaceae. Pairwise comparisons and sequence identity between *Hyoscyamus niger* and five solanaceous chloroplast genomes for selected regions using the VISTA program. The Y-axis represents the % identity (50–100%) across the chloroplast genome. Coding and non-coding regions are marked in green and blue, respectively. Pink boxes indicate known (empty boxes) and novel (filled boxes) plastid regions used in the phylogenetic analyses in this study.

Variable intergenic plastid regions with potential use in phylogenetics have been identified in several angiosperms [17], [18]. However, the phylogenetic applicability of non-coding plastid sequences are difficult to predict because the variability of each marker differs across related angiosperm clades [19]. A few variable non-coding regions (*trnL-F*, *rps16-trnK, trnC-psbM, psbA-trnH*) and plastid genes (*rbcL. ndhF, atpB*) have been previously sequenced from species of the tribe Hyoscyameae (Figure 2, empty boxes). Phylogenetic studies based on these markers with extensive taxon sampling showed that the genus *Atropa* was sister to the rest of the genera of the tribe [5], [10]. Furthermore, *Anisodus*, *Hyoscyamus*, and *Physochlaina* were monophyletic genera [5], [10], while *Scopolia* was monophyletic [10] or paraphyletic with respect to *Przewalskia* [5]. In addition, the genera *Physochlaina*, *Przewalskia* and *Scopolia* formed a well-supported clade [5], [10], but the relationships of this clade with the remaining genera of the tribe (*Hyoscyamus*, *Archihyoscyamus*, *Anisodus* and *Atropanthe*) were essentially unresolved due to low statistical support.

**Figure 3:**
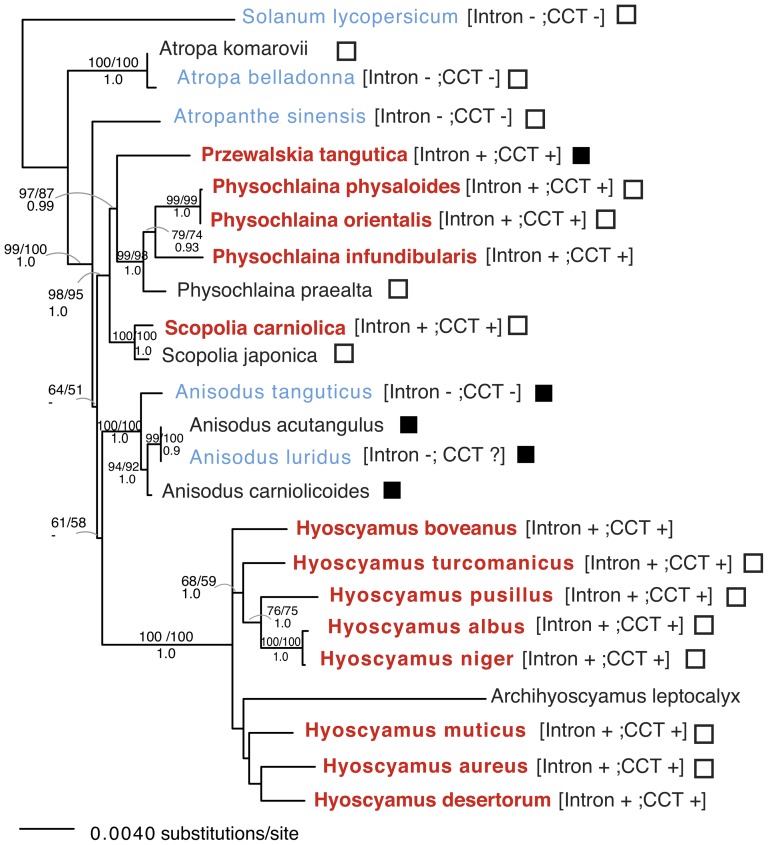
Maximum Likelihood phylogenetic tree of the tribe Hyoscyameae based on 10 chloroplast markers (11,610 bp). Taxa in red contain the *cox1* intron and CCT (co-conversion tract); taxa in light blue lack the *cox1* intron; taxa in black were not tested for the *cox1* intron. Filled and empty squares indicate taxa with and without an inversion in the intergenic region *trnC-psbM*, respectively. Numbers represent support values: 100 bootstrap (BS) replicates of ML analysis (top left), 1000 bootstrap replicates of MP analysis (top right) and posterior probabilities (PP) of Bayesian Inference (bottom). BS values and PP are shown when >50% and >0.9, respectively.

In this study, we identified three additional highly-variable plastid regions (*rps16-trnQ, ycf3-rps4, ndhF-rpl32*) for phylogenetic reconstruction within the tribe Hyoscyameae (Figure 2, filled boxes). Phylogenetic analyses based on individual (Figure S1) and concatenated plastid regions (Figure 3) were performed.

Trees based on individual plastid regions were not fully resolved due to the low phylogenetic signal of each molecular marker (Figure S1) and did not show strongly-supported conflicts among them (with one exception: *trnC-psbM*). Several genus-specific indels were found in the individual alignments, but none were shared by different genera; thus, they were not informative to elucidate relationships among genera of Hyoscyameae. The most variable regions for the tribe included *trnC-psbM, rps16-trnQ, ycf3-rps4* and *ndhF-rpl32* (Figure 2 and S1).

The plastid spacer *trnC-psbM* yielded a tree (Figure S1), where *Przewalskia* and *Anisodus* were sister taxa with strong bootstrap support (BS = 97%). After alignment screening, we detected a 10-nt polymorphic stretch shared by *Przewalskia* and *Anisodus*, which could be the result of a 10-nt inversion. We analyzed the surrounding sequence with Mfold [45] and detected a stem-loop forming structure with identical inverted repeats flanking the inversion and relatively high free energy (Figure S1). It is essential to recognize such microstructural changes because inversions like this can lead to robust but incorrect phylogenetic trees given its strong phylogenetic signal. The inversion event that took place within the plastid spacer *trnC-psbM* disrupted the site-wise homology across the 10 nt of the inversion. Thus, the dataset including the inversion violates the assumption of site-wise homology of a sequence alignment, yielding an incorrect phylogeny (Figure S1). The deletion of the 10-nt inversion significantly changed the topology of the *trnC-psbM* tree so that *Anisodus* and *Przewalskia* were no longer sister taxa (Figure S1). The inversion in the plastid spacer *trnC-psbM* (and its reversion) has occurred independently in several lineages within the Solanaceae [10], [46], [47]. The presence of the inversion in *Lycianthes* sp., *Solanum chilense* and *S. pennellii* and its absence in closely related taxa to the genus *Lycianthes* has been overlooked in an evolutionary study of the family Solanaceae, in which an incorrect plastid tree was found [10]. Recently, the inversion in the spacer *trnC-psbM* has been recognized through comparative genomic analysis in several species of the genus *Solanum* [46]. The 10-nt inversion is prone to homoplasy and should not be employed in phylogenetic studies. We removed the 10-nt inversion in all subsequent phylogenetic analyses.

We concatenated 10 plastid regions into a ∼11.6 kb data set (excluding the 10-nt inversion found in *trnC-psbM*) and analyzed it using a variety of phylogenetic approaches, including Maximum Likelihood (ML), Bayesian Inference and Maximum Parsimony (MP) (Figure 3). The data set consisted of 321 parsimony-informative sites. All three phylogenies were congruent and highly similar. Genera within the tribe were monophyletic with strong bootstrap support (Figure 3). A well-supported clade was formed by *Physochlaina* spp. + *Przewalskia*, which was sister to *Scopolia* spp. with strong support (BS>95; PP = 1.0). This grouping is also supported by seed characters [48], plastid markers [5], [10] and a nuclear gene [6]. The genus *Atropa* was sister to the remaining sampled Hyoscyameae, a relationship that was previously demonstrated by the presence of a retroposon in the nuclear gene GBSSI [6] and strong support in plastid phylogenies [5], [10]. The position of *Atropanthe sinensis*, as well as that of the genus *Anisodus*, was unresolved (Figure 3).

The eight species of *Hyoscyamus* analyzed were paraphyletic respect to *Archihyoscyamus leptocalyx*. *A. leptocalyx*, formerly known as *H. leptocalyx*, has been removed from the genus *Hyoscyamus* [8] based on flower and seed morphology, along with its unusual habitat in rock cliffs in western Asia [8], [48]. However, plastid molecular data (*ndhF*, *trnL-F* and the concatenated data set) showed that *A. leptocalyx* is embedded within a clade with species of *Hyoscyamus* [49] with strong bootstrap support (Figures 3 and S1). The taxonomic position of this species needs to be revisited using molecular data from the nuclear genome.

**Figure 4:**
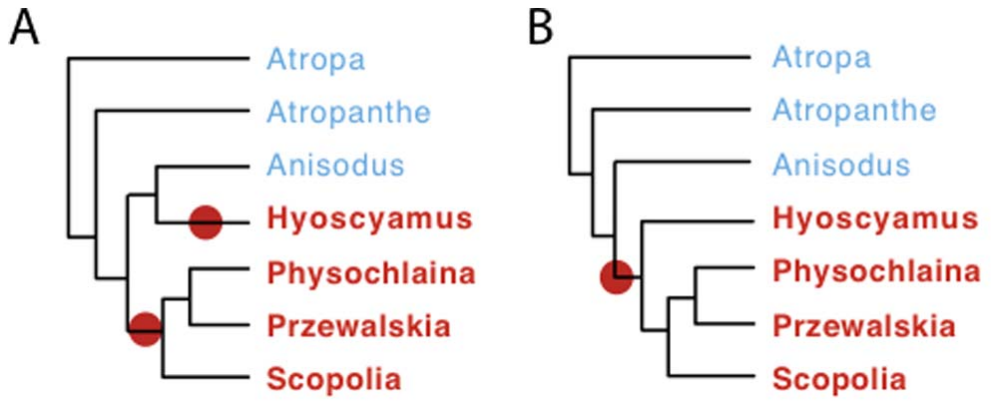
Alternative hypotheses for the evolution of the *cox1* intron and CCT in the tribe Hyoscyameae. Proposed evolutionary relationships within the tribe Hyoscyameae, showing intron acquisition (filled circle) by horizontal gene transfer. Taxa in red contain the *cox1* intron and CCT (co-conversion tract); taxa in light blue lack the *cox1* intron and CCT. **A.** Evolutionary hypothesis showing two independent intron acquisitions (homoplasious character) within the tribe. **B.** Evolutionary hypothesis based on a single intron acquisition (synapomorphy) within the tribe.

Based on the 11.6-kb plastid data set, we tested the possibility that the 10-nt inversion detected in the intergenic region *trnC-psbM* occurred independently in *Przewalskia* and *Anisodus* (as shown by the tree in Figure 3) or once in the common ancestor (in a constrained tree where *Przewalskia* and *Anisodus* were sister taxa). The AU test rejected (p = 7e-05) the sister relationship of *Przewalskia* and *Anisodus*, suggesting the parallel occurrence of this inversion (Figures 3–4) and showing it to be a homoplasious character.

#### Presence of the mobile *cox1* intron and a proposed evolutionary scenario for Hyoscyameae

In a previous study, we detected the presence of a group I intron in the mitochondrial gene *cox1* (cytochrome oxidase subunit 1) of all studied species of *Hyoscyamus* (9 species were analyzed), *Physochlaina* (3 species), *Przewalskia* (1 species) and *Scopolia* (1 species), while it was absent in the genera *Anisodus* (2 species), *Atropa* (3 species), and *Atropanthe* (1 species) [50] (Figure 3). Furthermore, intron-containing solanaceous taxa also had an 18 nt-signature in the flanking region of exon 2 (named the co-conversion tract - CCT), presumably obtained by gene conversion during the process of intron homing [50]. The *cox1* intron is highly mobile [51], [52] and has been horizontally transferred between two solanaceous lineages, *Mandragora* and Hyoscyameae [50]. The lack of this signature and the intron in the *cox1* gene in all the species of *Atropa*, *Atropanthe* and *Anisodus* analyzed (Figure 3) indicates that they never had the intron, instead of the unlikely alternative hypothesis suggesting the loss of both the intron and the signature [50]. Depending on the resolution of the phylogenetic position of *Anisodus* and *Atropanthe* within the tribe Hyoscyameae, one or two *cox1* intron acquisitions (Figure 4) may be proposed to explain the intron pattern observed (Figure 3).

The phylogenetic hypothesis shown in Figure 4A suggests two independent intron acquisitions: one in the ancestor of the genus *Hyoscyamus* and the other in the ancestor of the clade formed by *Przewalskia*, *Physochlaina* and *Scopolia*. Given that the *cox1* intron found in *Hyoscyamus* and the other Hyoscyameae are identical [50], one of the horizontal transfers of the intron may have occurred between members of the tribe; e.g. from *Physochlaina* to *Hyoscyamus*. Alternatively, based on the more parsimonious scenario of *cox1* intron evolution, we propose a second possible evolutionary history of the genera within the tribe Hyoscyameae, where the *cox1* intron was acquired once within the tribe (Figure 4B). Thus, the presence of the *cox1* intron is considered here a synapomorphy uniting five genera of the Hyoscyameae. An AU test based on the 11.6-kb chloroplast data set did not reject (p>0.05) a tree where the intron-containing taxa (*Hyoscyamus*-including *Archihyoscyamus-, Physochlaina, Przewalskia* and *Scopolia*) formed a monophyletic group (Figure 4B). The hypothetical tree topology shown in Figure 4B has not been recovered or rejected by any other study based on chloroplast markers [5], [10], nuclear genes [6], cytological studies [7] or morphological characters [1], [7], [53] and it is a valid evolutionary hypothesis for the tribe Hyoscyameae. Under either evolutionary scenario, the presence of the *cox1* intron and CCT is predicted for all species of *Hyoscyamus*, *Archihyoscyamus*, *Physochlaina* and *Scopolia*. In contrast, lack of the intron and CCT is expected for all species of *Anisodus*, *Atropa* and *Atropanthe* (Figure 4).

It is unclear why 11.6 kb of plastid sequence data were insufficient to completely resolve the relationships among genera of the tribe Hyoscyameae. The most likely explanation for the low phylogenetic resolution is a rapid diversification of the lineages within the tribe Hyoscyameae. If so, acquiring sufficient sequence data may help to resolve some of the polytomies [54], [55], [56]. It is possible that nuclear markers may aid in resolving evolutionary relationships, although the nuclear gene waxy [6] and the ribosomal spacers ITS1 and ITS2 [57] were not able to unravel the evolution of the tribe Hyoscyameae.

## Supporting Information

Figure S1 **Maximum Likelihood phylogenies of the tribe Hyoscyameae based on individual chloroplast regions using Garli.** All available plastid data from GenBank of species within Hyoscyameae were included. Numbers in parenthesis indicate length of the data set (left) and number of parsimony-informative characters (right). Bootstrap support values (above branches) are shown when >50%. The bar indicates the number of substitutions per site. *Solanum lycopersicum* (Solanum) was used as outgroup. New sequences from this study are in bold face. Different species of *Anisodus* (A.), *Hyoscyamus* (H.), *Physochlaina* (P.), and *Scopolia* (S.) are included. At the bottom, partial alignment of the intergenic region *trnC- psbM* with inverted repeats (arrows) and 10-nt inversion (underlined).
(PDF)

Table S1 **GenBank accession numbers for plastid markers used in the phylogenetic analyses in** **Figures 3** **and S1.**
(XLSX)
