# Supplementary material for: The Chloroplast Genome of Hyoscyamus niger and a Phylogenetic Study of the Tribe Hyoscyameae (Solanaceae)
Source: PLoS One. 2014 May 22;9(5):e98353. doi: 10.1371/journal.pone.0098353 (PMC4031233; doi:10.1371/journal.pone.0098353)

Figure S1. Maximum Likelihood phylogenies of the tribe Hyoscyameae based on individual chloroplast regions using Garli. All available plastid data from GenBank of species within Hyoscyameae were included. Numbers in parenthesis indicate length of the data set (left) and number of parsimony-informative characters (right). Bootstrap support values (above branches) are shown when >50%. The bar indicates the number of substitutions per site. *Solanum lycopersicum* (Solanum) was used as outgroup. New sequences from this study are in bold face. Different species of *Anisodus* (A.), *Hyoscyamus* (H.), *Physochlaina* (P.), and *Scopolia* (S.) are included. At the bottom, partial alignment of the intergenic region *trnC-psbM* with inverted repeats (arrows) and 10-nt inversion (underlined).

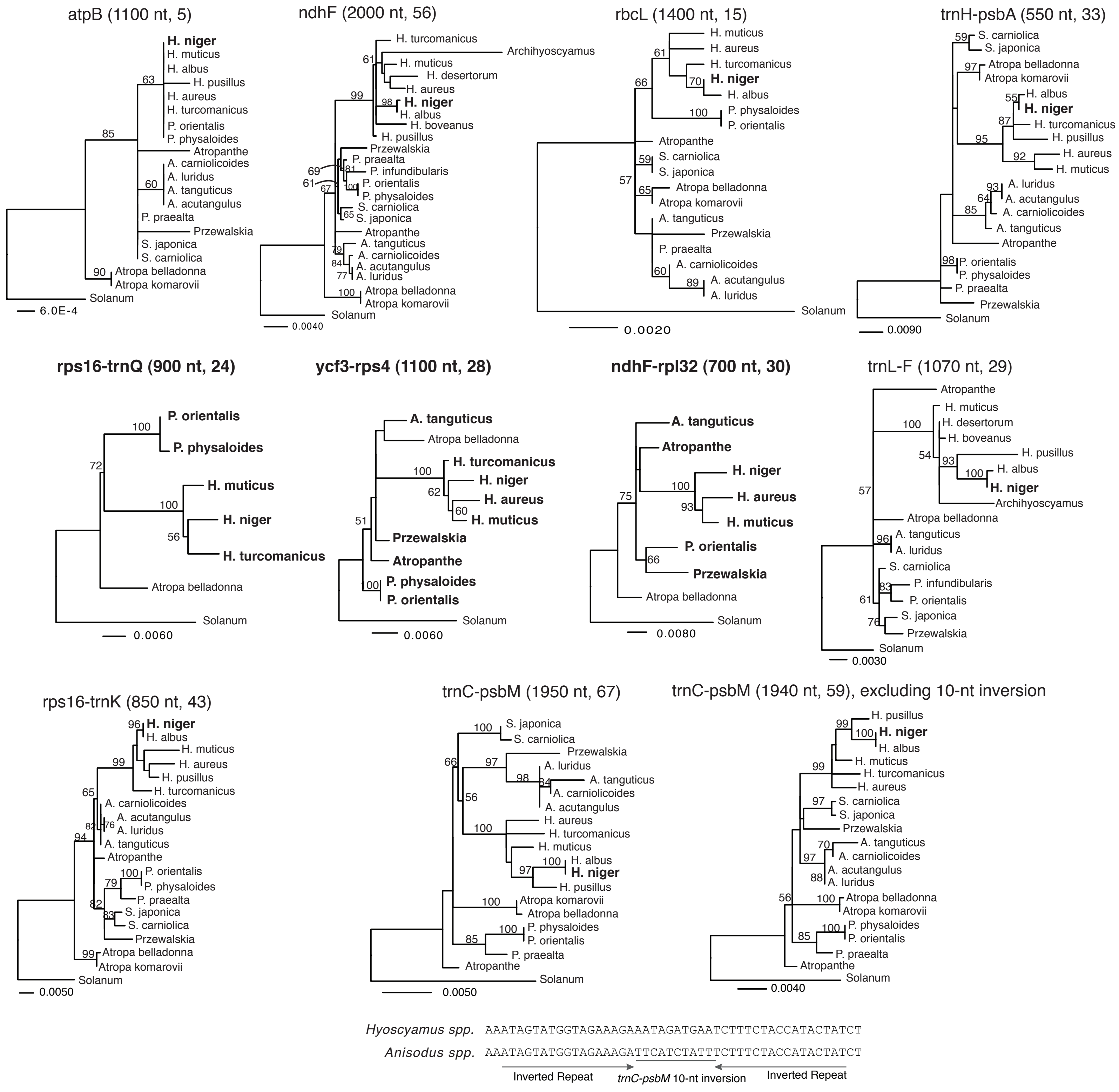

Supplement: Figure S1 — Maximum Likelihood phylogenies of the tribe Hyoscyameae based on individual chloroplast regions using Garli. All available plastid data from GenBank of species within Hyoscyameae were included. Numbers in parenthesis indicate length of the data set (left) and number of parsimony-informative characters (right). Bootstrap support values (above branches) are shown when >50%. The bar indicates the number of substitutions per site. Solanum lycopersicum (Solanum) was used as outgroup. New sequences from this study are in bold face. Different species of Anisodus (A.), Hyoscyamus (H.), Physochlaina (P.), and Scopolia (S.) are included. At the bottom, partial alignment of the intergenic region trnC- psbM with inverted repeats (arrows) and 10-nt inversion (underlined). (PDF) [file pone.0098353.s001.pdf]
